# Psychological impact of COVID‐19 on speech and language therapists working with adult dysphagia: A national survey

**DOI:** 10.1111/1460-6984.12654

**Published:** 2021-07-31

**Authors:** Rachel Rouse, Julie Regan

**Affiliations:** ^1^ Department of Clinical Speech & Language Studies Trinity College Dublin Dublin Ireland; ^2^ Speech and Language Pathology Department of Clinical Speech and Language Studies Trinity College Dublin Dublin Ireland

**Keywords:** dysphagia, speech and language therapists, psychological impact, COVID‐19

## Abstract

**Background:**

Speech and language therapists (SLTs) working with dysphagia have had to radically alter diagnostic and rehabilitation services during the severe acute respiratory syndrome coronavirus 2 (SARS‐CoV‐2) pandemic, hereafter referred to as coronavirus disease (COVID‐19). Given the aerosol‐generating procedures inherent in swallow assessment and interventions, these SLTs have also been particularly susceptible to virus exposure.

**Aims:**

To investigate the psychological impact of COVID‐19 on SLTs working with adult dysphagia across the Republic of Ireland and to identify the personal and professional factors associated with depression, anxiety, stress and post‐traumatic stress disorder (PTSD). To explore SLT perspectives regarding their experiences during the COVID‐19 pandemic.

**Methods & Procedures:**

A cross‐sectional 34‐item online survey was developed and piloted. The survey collected demographic details and professional factors and it incorporated the Depression, Anxiety, and Stress Scale—21 (DASS‐21) and the Impact of Event Scale—Revised (IES‐R). The survey also sought SLT perspectives regarding their experiences during the pandemic. It was distributed to Irish SLT managers, the Irish Association of Speech and Language Therapists (IASLT) and the Irish Dysphagia Special Interest Group (SIG) for dissemination.

**Outcomes & Results:**

A total of 94 SLTs working with adults with dysphagia across Ireland responded. In total, 60% of respondents screened positive for depression, anxiety, stress and/or PTSD. Based on the DASS‐21, 38% screened positive for depression (mean score = 8.0; SD = 6.3), 36% screened positive for anxiety (mean score = 6.5; SD = 6.0), and 49% screened positive for stress (mean score = 15.4; SD = 6.9). A total of 26% of respondents screened positive for PTSD (mean IES‐R total score = 22.6; SD = 16.0). Factors associated with depression, anxiety, stress and PTSD were young age (*p* = 0.002), limited clinical experience (*p* = 0.01) and not living with children (*p* = 0.02). A thematic analysis of SLT perspectives identified four main themes: ‘fear of COVID‐19 infection and transmission’, ‘uncertainty regarding policies and procedures’, ‘changes in SLT roles and responsibilities’ and ‘increased workload’.

**Conclusions & Implications:**

This study highlights the psychological impact of COVID‐19 on SLTs working with adults with dysphagia in Ireland and identifies SLTs who are at risk of depression, anxiety, stress and PTSD during the COVID‐19 pandemic. Findings may assist employers to identify staff who require psychological support and long‐term follow‐up during this pandemic and any future health emergencies.

**What this paper adds:**

**What is already known on the subject**

- Depression, anxiety, stress and PTSD are prevalent among nurses and other healthcare professionals internationally as a result of the COVID‐19 pandemic and associated factors have been identified in previous research. Despite this, no research has been conducted to establish the psychological impact of the first surge of COVID‐19 on SLTs working with dysphagia in relation to depression, anxiety, stress and PTSD and the associated factors.

**What this paper adds to existing knowledge**

- A total of 60% of SLTs working with adults with dysphagia in this survey presented with depression, anxiety, stress and/or PTSD. Associated personal and professional factors amongst respondents included young age, limited clinical experience and not living with children. The perspectives of SLTs on their experiences during the first surge of COVID‐19 are also explored.

**What are the potential or actual clinical implications of this work?**

- The findings from this study may assist SLT managers to identify SLTs who are at a higher risk of depression, anxiety, stress and PTSD to ensure appropriate support can be provided.

## INTRODUCTION

On 31 December 2019, the Wuhan Municipal Health Commission alerted the World Health Organisation (WHO) about an outbreak of cases of viral pneumonia in Wuhan, the capital of central China's Hubei province (WHO, 2020). The cause of this disease that was subsequently termed coronavirus disease (COVID‐19) was a virus named severe acute respiratory syndrome coronavirus 2 (SARS‐CoV‐2). The novel coronavirus outbreak was proclaimed a pandemic on 11 March 2020. The first confirmed case of COVID‐19 in the Republic of Ireland was announced on 29 February 2020, thereafter the number of confirmed cases and deaths escalated rapidly.

Individuals who were severely ill with the virus required hospitalization and, in some cases, intensive care unit (ICU) admission and mechanical ventilation (Kennelly et al., 2020). As a result, increased pressure and demand was placed on an already overstretched Irish health service, leading to a lack of essential resources such as ICU beds, ventilators and personal protective equipment (PPE) (Kennelly et al., 2020). Following the first wave of the pandemic, healthcare workers (HCWs), including speech and language therapists (SLTs), accounted for 8293 of the 25,960 (32%) confirmed cases reported as of 6 July 2020 (Health Protection Surveillance Centre, 2020). As a result of HCWs taking time off to self‐isolate and recover from the virus themselves, staffing issues arose, contributing to the increased pressure on the health service. Other workforce issues included redeployment of HCWs, particularly SLTs, to other roles or services to address the surge of COVID‐19‐positive patients and HCWs having to leave work for childcare purposes (Patterson et al., 2020).

The COVID‐19 pandemic was a time of intense anxiety for the entire public of Ireland (Burke et al., 2020). This was due to the scale and speed of COVID‐19 transmission, the potentially fatal outcomes associated with it, and the implementation of preventative measures such as the nationwide lockdown and stringent public health advice from the government and Health Service Executive (HSE) (Burke et al., 2020; Kelly, 2020). However, HCWs faced the added burden of working on the frontline, managing individuals who were COVID‐19 positive, although they themselves were not immune to the virus. Of the 8018 HCWs who had been infected with COVID‐19 up to 30 May, 88% contracted the virus in a healthcare setting (Kennelly et al., 2020).

Recent research investigating the psychological impact of COVID‐19 highlighted the significant psychological distress experienced by the general public of Ireland (Kelly, 2020) as well as HCWs (Maunder, 2004; Shechter et al., 2020; Spoorthy et al., 2020). A study by Hyland et al. (2020) reported that depression and anxiety were common among the Irish population during the pandemic; however, rates of psychological distress among HCWs were significantly higher (Kelly, 2020). A study of UK intensive care workers found that 28% had symptoms of post‐traumatic stress disorder (PTSD) as a result of COVID‐19 (Dykes et al., 2021). Another study of HCWs managing patients with COVID‐19 in five major hospitals across Singapore and India found that 96 (11%) participants screened positive for depression, 142 (16%) for anxiety, 47 (5%) for stress and 67 (7%) for clinical concern of PTSD (Chew et al., 2020). A study by Tan et al. (2020) investigated the psychological impact of COVID‐19 on medical HCWs such as physicians and nurses and non‐medical HCWs such as allied health care professionals treating patients with COVID‐19 in two major institutions in Singapore. The primary findings of this study included the incidence of depression, stress, anxiety and PTSD among both medical and non‐medical HCWs and the higher rate of anxiety among non‐medical HCWs (21%) compared with medical HCWs (11%). Also, higher Depression, Anxiety, and Stress Scale—21 (DASS‐21) and Impact of Event Scale—Revised (IES‐R) mean scores were observed in non‐medical HCWs. This evidence suggests the possibility that SLTs, as non‐medical HCWs treating COVID‐19 positive individuals, are susceptible to psychological distress.

SLTs have a central role in the multidisciplinary management of people with dysphagia. Clinical and instrumental dysphagia evaluations can include components such as cough testing, whereas dysphagia interventions often consist of swallowing and cough rehabilitation tasks. These are considered AGPs (Freeman‐Sanderson et al., 2021) as they involve the mucous membranes of the upper airway which have the highest viral load in the body and can elicit a cough (Miles et al., 2020). SLTs also risk exposure to saliva and to respiratory droplets and aerosols generated during dysphagia rehabilitation (Kimura et al., 2020). Recently, this has increased SLT risk of COVID‐19 infection and transmission which may contribute to psychological distress (Schindler et al., 2020). SLT dysphagia management practices therefore needed to be modified during the pandemic for the protection of SLTs and their patients (Miles et al, 2020).

Professional associations such as the Irish Association of Speech and Language Therapists (IASLT), the Royal College of Speech and Language Therapists (RCSLT) and the European Society for Swallowing Disorders (ESSD) developed guidelines for dysphagia management during COVID‐19 (IASLT, 2020; RCSLT, 2020a; Schindler et al., 2020). Based on these guidelines as well as local policies, SLTs received guidance in terms of risk of face to face versus indirect or virtual dysphagia evaluation and treatment. Components of the face‐to‐face clinical swallow evaluation including the orofacial exam were deemed medium to high risk and adjunct tests such as cough reflex testing were paused during the first wave of the pandemic (Miles et al, 2020). Instrumental examinations including fibreoptic endoscopic evaluation of swallowing were temporarily stopped (ENT UK, 2020). Furthermore, evidence‐based dysphagia interventions, such as expiratory muscle strength training, were avoided given transmission risk. Furthermore, there was inconsistent guidance supporting dysphagia assessment as an AGP (Bolton et al., 2020). All these issues contributed to uncertainty, and likely anxiety, amongst SLTs working with dysphagia during the pandemic (Chadd et al., 2021).

Previous research has examined professional (e.g., redeployment) and personal factors contributing to psychological distress amongst HCWs during COVID‐19. Zheng et al. (2020), Wanigasooriya et al. (2020) and Mattila et al. (2021) found variables such as PPE, well‐being support, level of exposure to moral dilemmas at work, relationship quality with family, age and city of residence influenced psychological well‐being. As SLTs are involved in the management of patients with COVID‐19, it is likely that both personal and professional factors also impact their psychological well‐being.

To the authors’ knowledge, the psychological impact of COVID‐19 on SLTs working with adults with dysphagia has not been evaluated to date. This study aims to examine the psychological impact of the first surge of COVID‐19 on SLTs working with adults with dysphagia and to identify the personal and professional factors associated with depression, anxiety, stress and PTSD among these SLTs. It also sought to explore SLT perspectives on their experiences working with adults with dysphagia in Ireland during the first surge of COVID‐19.

## METHODS

Ethical approval for the study was obtained from the School of Linguistics, Speech and Communication Sciences Research Ethics Committee, Trinity College Dublin.

### Study design

The study used a mixed‐methods design for the elaboration and development of research findings (Greene et al., 1989). An online cross‐sectional web‐based survey method was chosen as it facilitated timely, anonymous and cost‐effective data collection in a safe manner given the context of the pandemic (Kelley et al., 2003).

### Participants

#### Eligibility criteria

Respondents’ eligible for inclusion were qualified SLTs working with adults with dysphagia in the Republic of Ireland during the first surge of COVID‐19 (March–June 2020). Only SLTs in the Republic of Ireland were eligible for inclusion and it was a requirement that the SLTs included in the study worked during the first surge of COVID‐19. SLTs working with paediatric dysphagia were excluded as there are few SLTs working in the area of paediatric dysphagia in Ireland at present and COVID‐19 had less of an impact in the paediatric population. Hence, a subgroup analysis would not have been feasible.

#### Survey development

A 34‐item anonymous survey (10–15 min completion time) was designed using Qualtrics online software (Qualtrics Labs Inc., Provo, UT, USA) with reference to the frameworks and guidelines proposed by Tsang et al. (2017) and Boparai et al. (2018) (see Appendix B). The Tailored Research Method (Dillman, 2000) was also referred to during survey development in order to reduce measurement errors and maximize response rates by improving the visual appeal of the survey. The survey meets the criteria of the recently published Checklist for Reporting of Survey Studies (CROSS) (Sharma et al., 2021).

The survey comprised 33 closed questions in multiple choice and matrix table format and one open question in which SLTs were asked to reflect on their experiences during COVID‐19. Although closed questions limit the response options for participants, the survey consisted mainly of closed questions as open‐ended questions are more time‐consuming and difficult to code and score considering the project timeframe. The forced completion setting was not applied to any of the survey items as the researchers did not want participants to feel compelled to respond to survey items of a sensitive nature. Survey items were established by a thorough review of the literature and the COVID‐19 SLT practice guidelines developed by the IASLT, RCSLT and ESSD (IASLT, 2020; RCSLT, 2020a; Schindler et al., 2020). The survey was piloted with four SLTs registered with the Health & Social Care Professionals Council (CORU) to detect any issues that may have surfaced after publication of the survey. Feedback was sought on ease of completion, clarity and relevance of questions, survey length and adequacy of response options, and comments and suggestions were encouraged. The survey was refined based on the feedback provided.

#### Survey content and administration

The final survey consisted of three main components, which were designed to determine the psychological impact of COVID‐19 and to identify any associated personal or professional factors:

- *Personal factors* included in the survey were selected with reference to previous pandemic research in other healthcare disciplines (Mattila et al., 2021; Wanigasooriya et al., 2020; Zheng et al., 2020). Factors included were living with children, living with someone who could be severely affected by COVID‐19, marital status, age and work organization.
- *Psychological impact*: The second section of the survey comprised the Depression, Anxiety, and Stress Scale (DASS‐21; Lovibond, 1995) and the Impact of Event Scale—Revised (IES‐R; Weiss & Marmar, 1997). The DASS‐21 is a validated screening instrument developed by the University of New South Wales, Australia, that provides robust measures of the emotional states of depression, anxiety and stress. This instrument consists of three sets of self‐report scales with each of the three sets containing seven items. Scores for each of the three constituents were calculated and multiplied by two. Participants with scores of > 9, > 7 and > 14 screened positive for depression, anxiety and stress, respectively (Chew et al., 2020). Participants were regarded as mild, moderate, severe or extremely severe depending on depression, anxiety and stress subscales scores. The DASS‐21 is a tool already used in COVID‐19 research to explore the psychological impact of COVID‐19 on other HCWs and to examine the association between psychological outcomes and physical symptoms among HCWs during the COVID‐19 pandemic (Chew et al, 2020; Tan et al., 2020).

The IES‐R is a 22‐item self‐report scale that includes three subscales (Intrusion, Avoidance and Hyperarousal) that measure subjective distress triggered by traumatic events. Respondents were required to rate items on a five‐point scale to generate a total score and subscale scores. Participant scores were graded in terms of severity from normal (0–23), mild (24–32), moderate (33–36) to severe (> 37) psychological impact (Chew et al., 2020). This study utilized a cut‐off score of ≥ 33 to classify PTSD as a probable diagnosis (Creamer et al., 2003). A strength of the IES‐R is that it is associated with the Diagnostic and Statistical Manual of Mental Disorders (DSM‐IV) criteria for PTSD. The IES‐R was also employed in recent COVID‐19 research (Tan et al., 2020; Chew et al, 2020). It has also been incorporated in surveys used in COVID‐19 research by Wanigasooriya et al. (2020) and Dykes et al. (2021) assessing the psychological impact of HCWs during the pandemic. Both the DASS‐21 and the IES‐R were adapted to better suit the time period in which the survey was published. ‘During the past seven days’ was substituted with ‘during the COVID‐19 pandemic’.

- *Professional factors*: Questions in this section sought information on clinical and instrumental dysphagia evaluation practices, dysphagia rehabilitation, tracheostomy, PPE and the availability of psychological support in participant settings. These factors were informed by previous COVID‐19 research (Mattila et al., 2021; Zheng et al., 2020; Wanigasooriya et al., 2020).

#### Recruitment/dissemination

Sample size determination was not feasible as there is an undefined number of SLTs working with adults with dysphagia in Ireland during the COVID‐19 pandemic. Snowball sampling methodology was used to recruit participants as it is a quick and cost‐effective method of sampling that often results in increased cooperation (Ungvarsky, 2020). An executive officer based in the local university setting acted as a gatekeeper for the study to prevent sampling bias. On 30 June 2020, an email advertising the survey was sent by the gatekeeper to the Irish SLT Managers Group for dissemination to staff. Invitation emails containing the survey link were also forwarded to organizations such as the IASLT and the Irish Dysphagia SIG. The survey link was posted on Twitter by the gatekeeper, the IASLT and the Department of Clinical Speech and Language Studies (Borgmann et al., 2015). A reminder email was sent to the gatekeeper on 18 August 2020 to be forwarded to the IASLT and the Irish Dysphagia SIG with the aim of maximizing the response rate.

#### Data analysis

Data were downloaded from Qualtrics and exported into IBM SPSS 26.0 (IBM Corp, 2019) for analysis. For analysis purposes, data were stored in a double‐encrypted and password‐protected file, accessible only to the researchers. All survey data, irrespective of whether or not the participant completed the survey in full, were included in the analysis. Descriptive statistics were used to analyse quantitative data generated by closed questions. IBM SPSS Statistics was utilized for the performance of statistical analyses. In order to conduct inferential statistical tests, participants who screened positive for depression, anxiety, stress and/or PTSD were given a binary code of 1 and all other participants were given a code of 0. Inferential statistics such as *χ* ^2^ tests of association were used to formulate the interdependence between various personal and professional variables and psychological scale scores. Mann–Whitney *U*‐tests were also used to compare the differences in various personal and professional variables between those who screened positive for depression, anxiety, stress and/or PTSD and those who did not.

The final question of the survey required participants to take a moment to reflect on their experiences during COVID‐19. Participant responses to this question were downloaded and exported into a Microsoft Word document wherein qualitative thematic analysis was conducted following the phases outlined by Braun and Clarke (2006). In‐depth readings of the free‐text responses were completed in order to familiarize the researcher with the content of the data. The initial thoughts of the researcher and concepts and phrases of interest within the data were noted. Themes were generated inductively from the data. Initial codes were created with the use of thematic networks and the coded data extracts were categorized into themes that address the research question. Themes were then revised and defined depending on how accurately they reflect the meanings apparent in the data and the adequacy and quality of supporting data. Themes were named appropriately depending on the data captured within them. An analytical narrative encompassing extracts from the raw data was provided for each individual theme (Nowell et al., 2017).

## RESULTS

In total, 99 responses to the survey were received. A response rate could not be calculated as it was not possible to determine the total number of potential respondents due to the use of snowball sampling methodology (Andrew et al., 2017). Five responses were excluded due to not proceeding with survey (*n* = 1); not meeting inclusion criteria; or not providing information on eligibility (*n* = 4). A total of 94 responses were eligible for analysis; 83 were completed in full. Completion rates for each survey item are shown in Appendix A.

### Participant demographics

The demographic characteristics and personal and professional factors relating to the participants of this study are summarized in Table 1. The largest number of survey respondents practice in Leinster (66.3%, *n* = 61/92), with the next highest number (20.65%, *n* = 19/92) working in Munster. The remainder work in Connacht or Ulster (Donegal, Cavan, Monaghan). Over one‐third of respondents (35%) were redeployed during the COVID‐19 pandemic. Table 1 provides a description of the various posts to which SLTs were redeployed. The 14% of participants (*n* = 13/92) who selected ‘other’ reported that they were redeployed to carry out COVID‐19 testing or administrative work. The results suggest that most of the respondents work for public organizations (87%, *n* = 80/92). Other types of organizations that respondents worked for included voluntary bodies, semi‐state‐funded organizations and a registered charity (7%, *n* = 6/92). Other reported SLT work settings that were not accounted for in the survey answer options were disability services and rehabilitation services.

**TABLE 1:** Participant demographics and personal and professional factors

Characteristic		*n* (%)
Gender (*n* = 92)	Female	89 (97)
	Male	3 (3)
Age group (years) (*n* = 92)	21–30	38 (41)
	31–40	40 (43)
	41–50	11 (12)
	51–60	3 (3)
Marital status (*n* = 92)	Single	31 (34)
	Married or cohabitating	61 (66)
Years of experience (*n* = 92)	0–5	32 (35)
	6–10	24 (26)
	11–20	29 (32)
	21–30	6 (7)
	31–40	1 (1)
Work status (*n* = 91)	Working routinely as SLT	88 (97)
	On leave	1 (1)
	Redeployed	2 (2)
Redeployment (*n* = 92)	To another ward as SLT	7 (8)
	To another service as SLT	9 (10)
	To support other healthcare staff	3 (3)
	Other	13 (14)
	Never redeployed	60 (65)
Work organization (*n* = 92)	Private	6 (7)
	Public	80 (87)
	Other	6 (7)
Size of SLT department (*n* = 92)	1–5 SLTs	48 (52)
	6–10 SLTs	18 (20)
	> 10 SLTs	26 (28)
Tracheostomy training (*n* = 92)	Yes	37 (40)
	No	55 (60)
Living with children (*n* = 92)	Yes	29 (32)
	No	63 (68)
Living with someone who could be severely affected by COVID‐19 (*n* = 91)	Yes	18 (20)
	No	73 (80)

Abbreviation: SLT, speech and language therapist.

### Psychological impact

**FIGURE 1:**
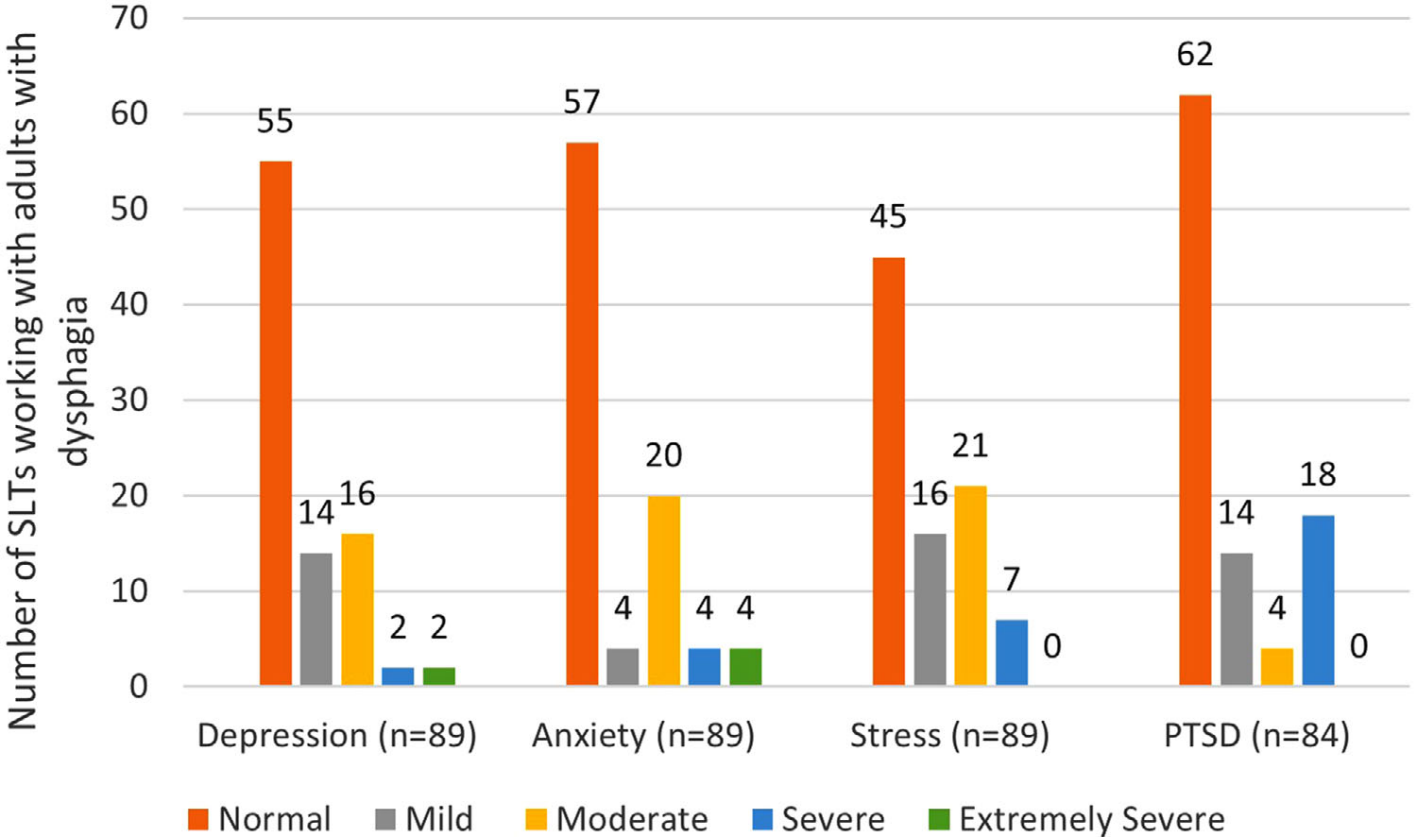
Prevalence and severity of depression, anxiety, stress and PTSD among SLTs working with adults with dysphagia in Ireland, measured by the DASS‐21 (depression, anxiety and stress) and IES‐R (PTSD). On the DASS‐21 depression subscales, scores of 10–13 = mild, 14–20 = moderate, 21–27 = severe and 28–42 = extremely severe. On the DASS‐21 anxiety subscales, scores of 8–9 = mild, 10–14 = moderate, 15–19 = severe and 20–42 = extremely severe. On the DASS‐21 stress subscales, scores of 15–18 = mild, 19–25 = moderate, 26–33 = severe and 34–42 = extremely severe. On the IES‐R, total scores of 24–32 = mild, 33–36 = moderate and > 37 = severe [Color figure can be viewed at wileyonlinelibrary.com]

A total of 53 respondents in total (60%, *n* = 53/89) screened positive for depression, anxiety and/or stress on the DASS‐21. Of these 53 participants, 19 (36%) had a combination of depression, anxiety and stress. A total of 49 of these participants proceeded to complete the IES‐R, with 21 (43%, *n* = 21/49) also screening positive for PTSD. A total of 22 participants (26%, *n* = 22/84) scored > 33 on the IES‐R, indicating PTSD as a probable diagnosis. Of the 84 SLTs working with dysphagia in Ireland who completed both psychological scales, 50 SLTs (60%, *n* = 50/84) screened positive for depression, anxiety, stress and/or PTSD as a result of the COVID‐19 pandemic. The prevalence and severity of depression, anxiety, stress and PTSD among SLTs working with adults with dysphagia during COVID‐19 pandemic are displayed in Figure 1.

The mean DASS‐21 depression, anxiety and stress subscale scores are presented in Table 2, along with the mean IES‐R total score and the mean intrusion, avoidance and hyperarousal subscale scores.

**TABLE 2:** Mean DASS‐21 and IES‐R scores

		*N*	Mean score	SD
DASS‐21	Depression	89	8.02	6.31
	Anxiety	89	6.52	5.96
	Stress	89	15.39	6.88
IES‐R	IES total	84	22.61	16.04
	Intrusion	84	1.12	0.96
	Avoidance	84	1.02	0.84
	Hyperarousal	84	0.96	0.88

*Notes*: Cut‐off scores > 9, > 7 and > 14 signify a positive screen for depression, anxiety and stress, respectively. A total IES‐R cut off score of ≥ 33 signifies PTSD as a probable diagnosis.

Abbreviation: DASS‐21, Depression, Anxiety, and Stress Scale—21; and IES‐R, Impact of Event Scale—Revised.

### Personal and professional factors associated with depression, anxiety, stress and PTSD

#### Personal factors

Personal and professional factors associated with psychological scale scores are presented in Table 3. Personal factors associated with a positive score for depression, anxiety, stress and PTSD included young age and not living with children (Table 3). The Mann–Whitney *U*‐test revealed a statistically significant difference between the median age group of SLTs who did not screen positive for psychological distress (median = 31–40 years) and the median age group of those that did (median = 21–30 years), indicating that younger participants were more likely to screen positive for psychological distress.

**TABLE 3:** Personal and professional variables associated with depression, anxiety, stress and/or PTSD

Variable	Result
*Personal factors*	
**Living with children** Number of SLTs living with children who screened positive for psychological distress: 11/50 Number of SLTs not living with children who screened positive for psychological distress: 39/50	*χ*^2^ (1, *N* = 84) = 5.83 *p* = 0.02*
**Living with someone who could be severely affected by COVID‐19** Number of SLTs living with someone who could be severely affected by COVID‐19 who screened positive for psychological distress: 10/49 Number of SLTs not living with someone who could be severely affected by COVID‐19 who screened positive for psychological distress: 39/49	*χ*^2^ (1, *N* = 83) = 0.00 *p* = 0.98
**Marital status** Number of married/cohabiting SLTs that screened positive for psychological distress: 31/50 Number of single SLTs that screened positive for psychological distress: 19/50	*χ*^2^ (1, *N* = 84) = 1.94 *p* = 0.16
**Age group** Median age of those who screened positive for psychological distress: 21–30 years Median age of those who did not screen positive for psychological distress: 31–40 years	*U* (*N* = 34, *N* = 50) = 534.00, *z* = –3.12 *p* = 0.002*
**Work organization** Number of SLTs who work for a private organization that screened positive for psychological distress: 1/50 Number of SLTs who work for a public organization that screened positive for psychological distress: 47/50 Number of SLTs who chose ‘other’ that screened positive for psychological distress: 2/50	*χ*^2^ (2, *N* = 84) = 1.95 *p* = 0.38 (> 20% of cells have expected count < 5)
	
*Professional factors*	
**Years of clinical experience** Median years of clinical experience of SLTs who screened positive for psychological distress: 6–10 years Median years of clinical experience of SLTs who did not screen positive for psychological distress: 11–20 years	*U* (*N* = 34, *N* = 50) = 556.50, *z* = –2.80 *p* = 0.01*
**Tracheostomy training** Number of SLTs who had tracheostomy training that screened positive for psychological distress: 20/50 Number of SLTs who have not had tracheostomy training that screened positive for psychological distress: 30/50	*χ*^2^ (1, *N* = 84) = 0.03 *p* = 0.87
**Redeployment** Number of SLTs who were redeployed that screened positive for psychological distress: 19/50 Number of SLTs who were not redeployed that screened positive for psychological distress: 31/50	*χ*^2^ (1, *N* = 84) = 0.06 *p* = 0.80
**Management of COVID‐19 patients with a tracheostomy** Number of SLTs that managed COVID‐19 patients with a tracheostomy that screened positive for psychological distress: 11/50 Number of SLTs that did not manage COVID‐19 patients with a tracheostomy that screened positive for psychological distress: 39/50	*χ*^2^ (1, *N* = 84) = 1.45 *p* = 0.23
**Provision of PPE training by employer** Number of SLTs whose employers’ provided PPE training that screened positive for psychological distress: 47/49 Number of SLTs whose employers did not provide PPE training that screened positive for psychological distress: 2/49	*χ*^2^ (1, *N* = 83) = 0.14 *p* = 0.71 (> 20% of cells have expected count < 5)
**Acquisition of a buddy system for the placement and removal of PPE** Number of SLTs with buddy system who screened positive for psychological distress: 17/48 Number of SLTs without buddy system who screened positive for psychological distress: 10/48 Number of SLTs that did not always have a buddy system that screened positive for psychological distress: 21/48	*χ*^2^ (2, *N* = 80) = 0.15 *p* = 0.93
**Provision of psychological support from employer** Number of SLTs whose employers provided psychological support that screened positive for psychological distress: 33/49 Number of SLTs whose employers did not provide psychological support who screened positive for psychological distress: 16/49	*χ*^2^ (1, *N* = 83) = 0.10 *p* = 0.75
**Consideration of dysphagia as an AGP in participant work setting** Number of SLTs whose work setting considered dysphagia as an AGP that screened positive for psychological distress: 29/49 Number of SLTs whose work setting did not consider dysphagia as an AGP that screened positive for psychological distress: 14/49 Number of SLTs who were unsure whether work setting considered dysphagia as an AGP that screened positive for psychological distress: 6/49	*χ*^2^ (2, *N* = 82) = 0.60 *p* = 0.74
**Assessment of adults with COVID‐19 infection** Number of SLTs that assessed patients with COVID‐19 who screened positive for psychological distress: 36/50 Number of SLTs that did not assess patients with COVID‐19 who screened positive for psychological distress: 14/50	*χ*^2^ (1, *N* = 84) = 0.50 *p* = 0.48
**Inclusion of an orofacial examination during clinical swallow evaluations** Number of SLTs that included an orofacial examination who screened positive for psychological distress: 20/50 Number of SLTs that did not include an orofacial examination who screened positive for psychological distress: 24/50 Number of SLTs that selected ‘N/A’ who screened positive for psychological distress: 6/50	*χ*^2^ (2, *N* = 84) = 1.29 *p* = 0.53
**Inclusion of a voluntary cough assessment during clinical swallow evaluations** Number of SLTs that included a voluntary cough assessment who screened positive for psychological distress: 6/50 Number of SLTs that did not include a voluntary cough assessment who screened positive for psychological distress: 39/50 Number of SLTs that selected ‘N/A’ who screened positive for psychological distress: 5/50	*χ*^2^ (2, *N* = 84) = 1.88 *p* = 0.39 (> 20% of cells have expected count < 5)
**Inclusion of laryngeal palpation during clinical swallow evaluations** Number of SLTs that included laryngeal palpation who screened positive for psychological distress: 13/50 Number of SLTs that did not include laryngeal palpation who screened positive for psychological distress: 33/50 Number of SLTs that selected ‘N/A’ who screened positive for psychological distress: 4/50	*χ*^2^ (2, *N* = 84) = 2.86 *p* = 0.24
**Inclusion of cervical auscultation in clinical swallow evaluations** Number of SLTs that included cervical auscultation who screened positive for psychological distress: 0/50 Number of SLTs that did not include cervical auscultation who screened positive for psychological distress: 45/50 Number of SLTs that selected ‘N/A’ who screened positive for psychological distress: 5/50	*χ*^2^ (2, *N* = 84) = 5.19 *p* = 0.08 (> 20% of cells have expected count < 5)
**Suspension of out‐patient dysphagia services in participants setting** Number of SLTs whose work settings suspended out‐patient dysphagia services that screened positive for psychological distress: 30/50 Number of SLTs whose work settings did not suspend out‐patient dysphagia services that screened positive for psychological distress: 7/50 Number of SLTs that selected ‘N/A’ that screened positive for psychological distress: 13/50	*χ*^2^ (2, *N* = 84) = 1.26 *p* = 0.53
**Ability to conduct swallowing intervention with adults with COVID‐19 when indicated** Number of SLTs who could carry out intervention with COVID‐19 patients that screened positive for psychological distress: 35/49 Number of SLTs who could not carry out intervention with COVID‐19 patients that screened positive for psychological distress: 14/49	*χ*^2^ (1, *N* = 82) = 0.03, *p* = 0.87

*Notes*: Psychological distress refers to depression, anxiety, stress and/or PTSD.

Abbreviation: AGP, aerosol‐generating procedure; PPE, personal protective equipment; PTSD, post‐traumatic stress disorder; and SLT, speech and language therapist.

#### Professional factors

In terms of professional factors, years of clinical experience was associated with positive psychological scale scores. Years of experience of SLTs who did not screen positive for psychological distress (median = 11–20 years) were greater than the years of experience of SLTs who did screen positive for psychological distress (median = 6–10 years). The Mann–Whitney *U*‐test indicated that this difference was statistically significant meaning that SLTs with less clinical experience were more likely to screen positive for psychological distress. No statistically significant findings were identified among the other personal and professional variables tested (Table 3).

#### SLT perspectives

**FIGURE 2:**
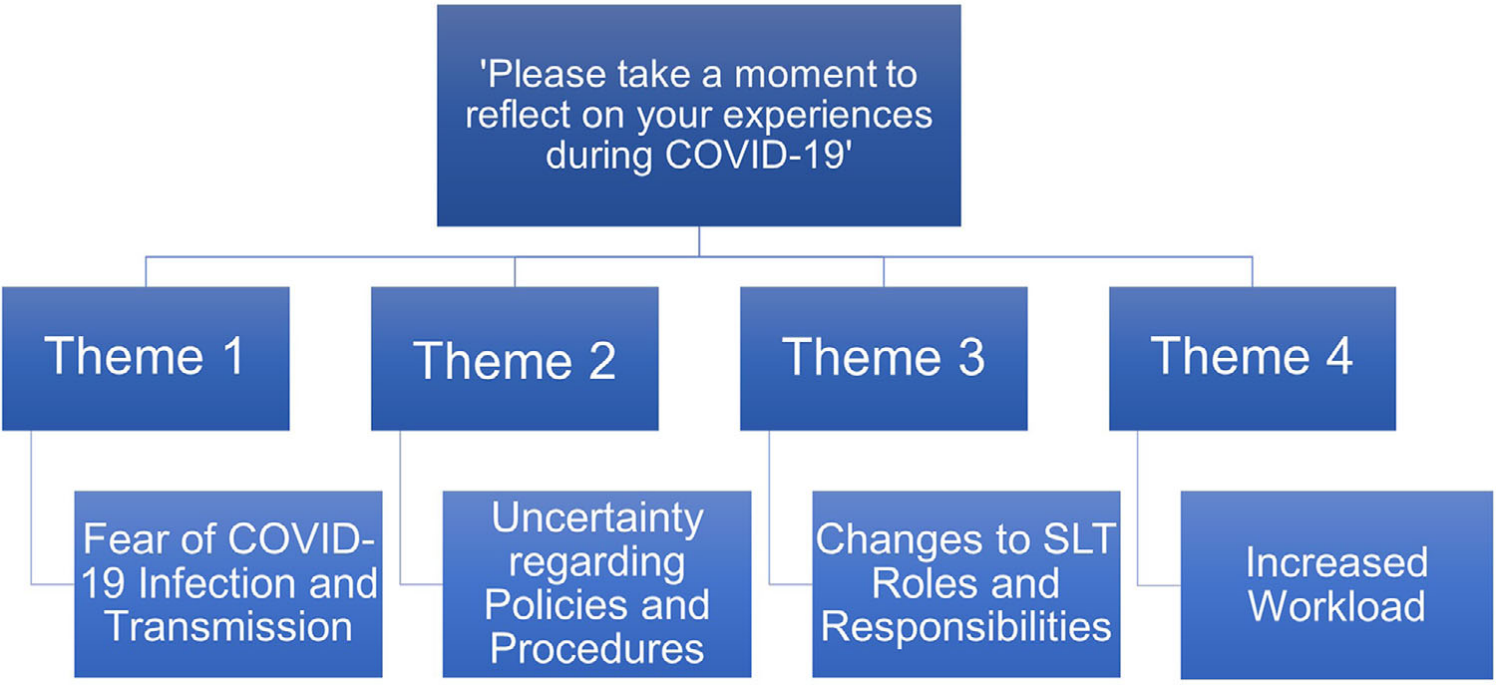
Themes identified within free‐text responses [Color figure can be viewed at wileyonlinelibrary.com]

Based on SLT perspectives, four main themes were identified through thematic analysis. The themes arising from the free‐text responses are summarized in Figure 2.

### Theme 1: Fear of COVID‐19 infection and transmission

From the perspective of SLTs working with adults with dysphagia in Ireland, the first wave of the COVID‐19 pandemic was a time of fear. Nine participants (18%, *n* = 9/50) reported feeling fearful of COVID‐19 infection and transmission whilst working during the COVID‐19 pandemic: ‘This fear was difficult to deal with at times.’ SLTs described a profound fear of becoming infected with the virus themselves: ‘today could be the day I get infected and end up in ICU’, and subsequently passing it on to patients or loved ones at home, particularly immunocompromised friends, or family members: ‘[It was an] awful time of worry about catching the virus and spreading it to patients or bringing it home.’ As a result, participants were required to avoid contact with loved ones, move out of their homes or organize alternative living arrangements: ‘I didn't see my family for months, I didn't get to go home. I didn't see my friends who I rely on so much. I didn't see my boyfriend as he has a condition that places him in the high‐risk category.’ While many respondents described a fear of COVID‐19 infection, three participants (6%, *n* = 3/50) reported that they had been infected with COVID‐19 with one SLT suffering with ‘significant post viral fatigue’ and another participant subsequently infecting his/her father which they reported as ‘highly impactful on wellness’.

### Theme 2: Uncertainty regarding policies and procedures

It appears that SLTs view their experiences working during the first surge of COVID‐19 as a time of uncertainty. Nine SLTs (18%, *n* = 9/50) described uncertainty concerning their specific roles and responsibilities as an SLT: ‘One of the most difficult aspects was discerning exactly what we should or should not be doing as SLTs’ as well as uncertainty regarding how best to manage patients during the COVID‐19 pandemic: ‘Management of patients with COVID[‐19] was complicated by … our own limited knowledge of COVID[‐19].’ It appears that this uncertainty arose from a lack of information at the beginning of the pandemic and an influx of information as time progressed: ‘Information overload from employer and professional bodies.’ Six SLTs (12%, *n* = 6/50) reported dissatisfaction in terms of the guidance provided by their organization and their governing body: ‘I don't feel our governing body did enough’; ‘Uncertainty and lack of leadership at times’; ‘Having large gaps in information about how to safely adapt our SLT service was very difficult.’ Also, three respondents (6%, *n* = 3/50) reported confusion and uncertainty regarding the consideration of dysphagia as an AGP: ‘IASLT and my organisation's policy regarding dysphagia assessments as an AGP differed’ with one SLT reporting that they felt ‘extremely frustrated’ due to the fact that dysphagia was not considered an AGP in their organization.

### Theme 3: Changes in SLT roles and responsibilities

It appeared that SLTs perceived their experiences of working during the first surge of COVID‐19 as a time of change. The free‐text responses reflect the many changes and modifications that were made in relation to the roles of SLTs and to SLT policies and procedures with participants’ roles changing entirely as a result of redeployment: ‘it was a sudden change in direction from my normal working day’. Six SLTs (12%, *n* = 6/50) reported that they were obligated to work remotely, limit or avoid face to face interactions with clients, reduce the length of time spent with clients, and adapt assessments and intervention in adherence with COVID‐19 restrictions. Five participants (10%, *n* = 5/50) reported feelings of guilt regarding their inability to provide the care that certain service users required: ‘[It] felt like we were not providing an adequate service to the vulnerable people that we support’; ‘I also felt that I couldn't provide the usual care that I usually would because of timing and assessment limitations.’

### Theme 4: Increased workload

Immunocompromised SLTs reported that they were required to work from home for their protection, not seeing clients directly. This increased the workload of SLTs not working remotely, intensifying their work environment. Also, staffing issues occurred as a result of the redeployment of SLTs, SLTs working remotely and numbers of staff on sick leave due to COVID‐19 infection: ‘I was off work with Covid‐19 very early on in the pandemic.’ Three SLTs (6%, *n* = 3/50) reported that they became close contacts of individuals who had the virus and were required to self‐isolate’: ‘I was exposed without correct PPE to a covid positive patient due to poor staff communication/documentation on one occasion and ended up quarantining my family and requiring testing.’ Staffing issues also occurred as a result of childcare issues with one participant reporting: ‘My husband and I had to manage childcare between us.’ As the global pandemic was not anticipated, the creation of clear plan was time consuming and intense: ‘The need to try and achieve a clear plan for our SLT department meant that a huge amount of extra work was required, often into evenings and weekends, and was exhausting and very stressful.’ SLTs also had to acquire new skills and knowledge to better manage clients during the pandemic increasing the cognitive demand on SLTs: ‘There was a huge volume of information and training to take on board.’ One participant described working during the COVID‐19 pandemic as a ‘real learning curve’. As well as staffing issues, education, training and adapting to change also increased the workload for SLTs hence reported feelings of exhaustion. Ten SLTs (20%, *n* = 10/50) reported that working during the COVID‐19 pandemic was ‘exhausting’, both physically and mentally: ‘my strongest feeling is tiredness’.

## DISCUSSION

Previous research has investigated the psychological impact of major health crises such as the COVID‐19 pandemic on HCWs globally. This was the first study to investigate the psychological impact of the first surge of COVID‐19 on SLTs working with dysphagia in the Republic of Ireland. Survey findings suggest the impact was substantial with 60% of respondents screening positive for depression, anxiety stress and/or PTSD. This study also found a significant association between a positive score for depression, anxiety, stress and/or PTSD, and personal and professional factors including young age, limited clinical experience and not living with children. The results of this study also illustrate that SLTs perceive their experiences of working with dysphagia during the first surge of COVID‐19 as a time of fear and uncertainty involving modifications to their roles and responsibilities and an increased workload.

Possible reasons behind the adverse psychological impact of COVID‐19 on SLTs working with dysphagia in Ireland and the negative perspectives of SLTs expressed include the highly transmissible nature of COVID‐19 and the risk of COVID‐19 infection faced by SLTs working with dysphagia. Another potential reason may be that SLTs are a small profession that are not as recognized as other professions working on the frontline. High levels of uncertainty regarding AGP policies and the best SLT management of patients with dysphagia during the COVID‐19 pandemic may also have contributed (Bolton et al., 2020). Another potential contributing factor may be the moral injuries resultant of SLTs not being able to provide their usual care and services to clients due to COVID‐19 restrictions (Greenberg et al., 2020). Reduced staffing levels leading to an increased workload for some SLTs as well as an influx of COVID‐19‐related information increased the mental load of SLTs which likely exacerbated psychological distress also. Finally, it is likely that the lack of guidance and information from governing bodies and management including a definition of an AGP and whether dysphagia assessments were considered AGPs contributed to the psychological distress of SLTs. Despite the many reasons for psychological distress during the COVID‐19 pandemic, many positive outcomes have emerged. Benefits such as improved colleague relationships, feelings of immense pride and enhanced teamwork were also reported. Another likely benefit of the pandemic is the advancement of telehealth in the field of speech and language therapy (Dimer et al., 2020).

The key findings of this study indicate that SLTs who do not live with children, which belong to a younger age group and/or have less clinical experience, may be more susceptible to depression, anxiety, stress and PTSD. Reasons for this may include the lack of clinical experience of younger newly qualified SLTs. It is probable that SLTs who began working prior to the unprecedented time of the COVID‐19 pandemic were familiarizing themselves with the policies and procedures of their new work setting until significant modifications were made in light of the virus. A possible reason that depression, anxiety, stress and/or PTSD were associated with SLTs not living with children is that the COVID‐19 pandemic was an isolating time for many individuals (Saltzman et al., 2020) and children provide significant social support.

No previous research evaluating the psychological impact of the first surge of COVID‐19 on SLTs were found. However, studies exploring the psychological impact of the first surge of COVID‐19 on HCWs from other countries were found. The mean IES‐R total score of SLTs in Ireland was more than twice as high as the mean IES‐R total score of the non‐medical health care personnel in Singapore involved in the 2020 study of Tan et al. (2020). Overall mean DASS‐21 scores and IES‐R scores were markedly higher among SLTs in the Republic of Ireland than HCWs in Singapore and India involved in a study by Chew et al. (2020). Potential reasons for the lower scores in other studies may include the provision of more psychological support to HCWs, better healthcare systems, lower rates of COVID‐19 infection and transmission, more ICU beds or increased access to PPE.

The IES‐R was also utilized in studies by Wanigasooriya et al. (2020) and Dykes et al. (2021) investigating the psychological impact of COVID‐19 on HCWs and intensive care workers in the UK. The rate of symptoms of PTSD among HCWs in the UK (24.5%) was similar to the rate of symptoms of PTSD among SLTs in Ireland (26%). The aforementioned study by Dykes et al. (2021) reported a mean total IES‐R score of 23, which is the same as the mean total IES‐R score reported for the SLTs involved in this study, indicating that, on average, SLTs in Ireland and intensive care workers in the UK received similar scores on the IES‐R.

Similar to a study by Mattila et al. (2021) investigating the associated factors of anxiety among hospital staff working during COVID‐19, young age was a factor associated with psychological distress among SLTs working with dysphagia in Ireland during the first surge of COVID‐19. Dissimilar to the findings reported by Wanigasooriya et al. (2020), the availability of adequate PPE, well‐being support and level of exposure to moral dilemmas at work were not associated with depression, anxiety, stress and PTSD among Irish SLTs. Possible reasons for this may be increased availability of PPE in Ireland in comparison with the UK, greater access to more effective psychological supports for HCWs in Ireland and fewer moral dilemmas within Irish work settings. This may be due to the significantly higher incident rates of COVID‐19 infection and transmission in the UK during the first wave of the pandemic, the approach taken by the UK government with regard to COVID‐19 restrictions and differing policies between Irish and UK work settings.

The key findings in this study are of clinical significance as the psychological well‐being of our profession needs to be protected. This study provides a profile of SLTs who may be at risk of depression, anxiety, stress and/or PTSD. The profile should aid SLT managers in the identification of vulnerable SLTs susceptible to psychological distress so that these SLTs can be adequately supported. Additionally, if our profession is supported, we are in a position to provide a high standard of service to people with dysphagia. The findings of this study emphasize the need for SLT managers to monitor and protect the psychological well‐being of all employees and particularly those who do not live with children and younger SLTs with fewer years of work experience. Potential psychological supports may include professional counselling services, peer support systems and guidance for leaders and managers supporting colleagues during the pandemic from national organizations such as RCSLT (2020b).

### Limitations

This study is not without limitations. Although the survey assessed whether participants lived with someone who could be severely affected by COVID‐19 infection, the survey did not query whether the participants themselves could be severely impacted by COVID‐19 infection or if participants became infected with COVID‐19 whilst working during the pandemic. These may have been potential factors associated with depression, anxiety, stress and PTSD. Also, the survey did not gather information on the psychological status of participants pre‐pandemic, for example, whether they experienced symptoms of psychological distress prior and whether they were requiring treatment for these symptoms. Finally, the chosen formulation of various survey items made data analysis difficult or impossible in some cases. For example, the association between the local policies of participant work settings for instrumental exams such as Fiberoptic Endoscopic Evaluation of Swallow (FEES) and depression, anxiety, stress, and PTSD could not be investigated due to the multiple‐choice matrix table format of the survey item.

## CONCLUSIONS

This study highlights the need for SLTs working with adults with dysphagia in the Republic of Ireland to be adequately supported during the COVID‐19 pandemic. SLTs belonging to younger age groups, who have fewer years of clinical experience and do not live with children, are at highest risk of depression, anxiety, stress and PTSD during the COVID‐19 pandemic. The key findings of this study provide SLT managers with an insight into the employees that may benefit from psychological supports, interventions and long‐term follow up during the remainder of this pandemic and in any future health emergencies. This study contributes to the evidence base supporting the need for health care professionals to be adequately supported during the COVID‐19 pandemic and acts as a basis for future research. Also, the findings of this study result in a better understanding of SLT needs and may impact the readiness of response to potential future health emergencies.

## CONFLICT OF INTEREST

None

## Supporting information

Supporting Information
