# Supplementary material for: Psychological impact of COVID‐19 on speech and language therapists working with adult dysphagia: A national survey
Source: Int J Lang Commun Disord. 2021 Jul 31;56(5):1037–52. doi: 10.1111/1460-6984.12654 (PMC8441712; doi:10.1111/1460-6984.12654)
Supplement: Supplementary file 1 — Supporting Information [file JLCD-56-1037-s001.docx]

**Appendix A. Completion rates for each survey item.**

**Appendix B. Survey**

Q1. Are you a qualified SLT working with adults with dysphagia in Italy or Ireland during the COVID-19 pandemic?

Yes

No

Section 1. Demographic Information

Q2. What gender do you identify as?

Female

Male

Other _____________________

Q3. What age group do you belong to?

21-30

31-40

41-50

51-60

over 60

Q4. Which province do you work in?

Province of __________________

Q5. Marital Status

Single

Married or cohabitating

Divorced or separated

Widowed

Q6. How long have you been working as an SLT?

0-5 years

6-10 years

11-20 years

21-30 years

31-40 years

over 40 years

Q7. What is your current work status?
 Working routinely as SLT

On leave

In quarantine

Redeployed

Q8. Were you redeployed during the pandemic, if yes where to?

To another ward as an SLT

To another service as an SLT

To support other healthcare staff (not working as an SLT)

Other __________________

I have never been redeployed

Q9. What type of organisation do you work for?

Private

Public

Academic setting

Other ___________________

Q10. What setting are you currently working in?

Hospital

Community Health Clinic

Residential Care Setting

Hospice – Palliative Care

Academic Setting

Other ________________

Q11. How many SLTs work in your department?

Insert number in the box provided

______________________________

Q12. Have you had tracheostomy training?

Yes

No

Q13. Do you live with any children?

Yes

No

Q14. Do you live with anyone who could be severely affected by COVID-19 (elderly, immunocompromised, etc.)?

Yes

No

Section 2. Psychological Scales

Q15. Adaptation of the Depression Anxiety Stress Scales-21 (Lovibond & Lovibond, 1995)

Please read each statement and choose either NEVER, SOMETIMES, OFTEN or ALMOST ALWAYS, depending on how much the statement applied to you during the COVID-19 pandemic. There are no right or wrong answers. Do not spend too much time on any statement.

The rating scale is as follows:

- Did not apply to me at all - NEVER

- Applied to me to some degree, or some of the time - SOMETIMES

- Applied to me to a considerable degree, or a good part of time - OFTEN

- Applied to me very much, or most of the time - ALMOST ALWAYS

| 1 | I found it hard to wind down | 0 1 2 3 |
| --- | --- | --- |
| 2 | I was aware of dryness of my mouth | 0 1 2 3 |
| 3 | I couldn't seem to experience any positive feeling at all | 0 1 2 3 |
| 4 | I experienced breathing difficulty (eg, excessively rapid breathing, breathlessness in the absence of physical exertion) | 0 1 2 3 |
| 5 | I found it difficult to work up the initiative to do things | 0 1 2 3 |
| 6 | I tended to over-react to situations | 0 1 2 3 |
| 7 | I experienced trembling (eg, in the hands) | 0 1 2 3 |
| 8 | I felt that I was using a lot of nervous energy | 0 1 2 3 |
| 9 | I was worried about situations in which I might panic and make a fool of myself | 0 1 2 3 |
| 10 | I felt that I had nothing to look forward to | 0 1 2 3 |
| 11 | I found myself getting agitated | 0 1 2 3 |
| 12 | I found it difficult to relax | 0 1 2 3 |
| 13 | I felt down-hearted and blue | 0 1 2 3 |
| 14 | I was intolerant of anything that kept me from getting on with what I was doing | 0 1 2 3 |
| 15 | I felt I was close to panic | 0 1 2 3 |
| 16 | I was unable to become enthusiastic about anything | 0 1 2 3 |
| 17 | I felt I wasn't worth much as a person | 0 1 2 3 |
| 18 | I felt that I was rather touchy | 0 1 2 3 |
| 19 | I was aware of the action of my heart in the absence of physical exertion (eg, sense of heart rate increase, heart missing a beat) | 0 1 2 3 |
| 20 | I felt scared without any good reason | 0 1 2 3 |
| 21 | I felt that life was meaningless | 0 1 2 3 |

Q16. Adaptation of the Impact of Event Scale - Revised (Weiss & Marmar, 1997)

Below is a list of difficulties people sometimes have after stressful life events. Please read each item, and then indicate how distressing each difficulty has been for you DURING THE COVID-19 PANDEMIC, which began in December 2019.

How much were you distressed or bothered by these difficulties?

Item Response Anchors are:

- Not at all;

- A little bit;

- Moderately;

- Quite a bit;

- Extremely.

|  | **Not at all** | **A little bit** | **Moderately** | **Quite a bit** | **Extremely** |
| --- | --- | --- | --- | --- | --- |
| Any reminder brought back feelings about it | 0 | 1 | 2 | 3 | 4 |
| I had trouble staying asleep | 0 | 1 | 2 | 3 | 4 |
| Other things kept making me think about it | 0 | 1 | 2 | 3 | 4 |
| I felt irritable and angry | 0 | 1 | 2 | 3 | 4 |
| I avoided letting myself get upset when I thought about it or was reminded of it | 0 | 1 | 2 | 3 | 4 |
| I thought about it when I didn’t mean to | 0 | 1 | 2 | 3 | 4 |
| I felt as if it hadn’t happened or wasn’t real | 0 | 1 | 2 | 3 | 4 |
| I stayed away from reminders about it | 0 | 1 | 2 | 3 | 4 |
| Pictures about it popped into my mind | 0 | 1 | 2 | 3 | 4 |
| I was jumpy and easily startled | 0 | 1 | 2 | 3 | 4 |
| I tried not to think about it | 0 | 1 | 2 | 3 | 4 |
| I was aware that I still had a lot of feelings about it, but I didn’t deal with them | 0 | 1 | 2 | 3 | 4 |
| My feelings about it were kind of numb | 0 | 1 | 2 | 3 | 4 |
| I found myself acting or feeling as though I was back at that time | 0 | 1 | 2 | 3 | 4 |
| I had trouble falling asleep | 0 | 1 | 2 | 3 | 4 |
| I had waves of strong feelings about it | 0 | 1 | 2 | 3 | 4 |
| I tried to remove it from my memory | 0 | 1 | 2 | 3 | 4 |
| I had trouble concentrating | 0 | 1 | 2 | 3 | 4 |
| Reminders of it caused me to have physical reactions, such as sweating, trouble breathing, nausea, or a pounding heart | 0 | 1 | 2 | 3 | 4 |
| I had dreams about it | 0 | 1 | 2 | 3 | 4 |
| I felt watchful or on-guard | 0 | 1 | 2 | 3 | 4 |
| I tried not to talk about it | 0 | 1 | 2 | 3 | 4 |

Section 3. Clinical/Instrumental Evaluation

Q17. During the pandemic were you asked to assess people with suspected dysphagia who were COVID-19 positive and/or had a suspected diagnosis of COVID-19?

Yes

No

Q18. Did you include an orofacial examination during clinical swallow assessments?

Yes

No

N/A

Q19. Did you include a voluntary cough assessment?

Yes

No

N/A

Q20. Did you assess laryngeal excursion with palpation during swallow evaluations?

Yes

No

N/A

Q21. Did you include cervical auscultation in swallow evaluations?

Yes

No

N/A

Q22. What was the local policy for instrumental exams on COVID-19 patients during the height of the pandemic?

|  | Not completing | When urgent | Completing routinely | No access to this instrumental exam |
| --- | --- | --- | --- | --- |
| FEES |  |  |  |  |
| VFS |  |  |  |  |
| High resolution manometry |  |  |  |  |
| Other |  |  |  |  |

Section 4. Rehabilitation

Q23. Were you able to carry out swallowing intervention with COVID-19 patients who required it?

No

Sometimes

In an adapted form

Yes

If yes, were you carrying out… (you can tick more than one option)

Direct rehabilitation

Compensatory Strategies

Other ____________________

Q24. Did your setting suspend outpatient dysphagia services?

Yes

No

N/A

Q25. During this pandemic did you assess or manage COVID-19 patients with a tracheostomy?

Yes

No

Skip To: End of Block If During this pandemic did you assess or manage COVID-19 patients with a tracheostomy? = No

Q26. Did you deflate the cuff of the tracheostomy tube in patients who were COVID-19 positive?

Yes

No

Q27. Did you carry out endotracheal suctioning in COVID-19 patients with a tracheostomy tube?

Yes

No

Q28. Were you involved in the proning of patients?

Yes

No

Section 5. Personal Protective Equipment

Q29. Did you always have the personal protective equipment (PPE) that you needed for assessing and treating COVID-19 patients?

Yes

No

If not, what equipment was limited (you can tick more than one option)

Gloves

Overalls

Shoe covers

FFP2/FFP3 masks

Surgical masks

Goggles

Hairnets

Q30. Did your employer provide PPE training?
 Yes

No

Q31. Did you have a buddy system for the placement and removal of PPE in your setting?

Yes

No

Not always

Q32. Was dysphagia considered an aerosol generating procedure (AGP) in your setting?

Yes

No

Unsure

Section 6. Psychological Impact

Q33. Has your employer provided psychological support?

Yes

No

Q34. Please take a moment to reflect on your experiences during COVID-19.

­­­­­­­______________________________________________________________________________________________________________________________________________________________________________________________________________________________________________________
